# Profiling Soil–Plant–Microbial Communities: DNA and Multi-Omics Techniques

**DOI:** 10.3390/genes17030303

**Published:** 2026-03-02

**Authors:** Shunlei Li, Claudia Chiodi, Carmelo Maucieri, Maria Cristina Della Lucia, Giulia Zardinoni, Samathmika Ravi, Andrea Squartini, Giuseppe Concheri, Gui Geng, Yuguang Wang, Piergiorgio Stevanato

**Affiliations:** 1 Department of Agronomy, Food, Natural Resources, Animals and Environment, University of Padova, Viale Università, 16, 35020 Legnaro, PD, Italy; shunlei.li@phd.unipd.it (S.L.); claudia.chiodi@unipd.it (C.C.); carmelo.maucieri@unipd.it (C.M.); mariacristina.dellalucia@unipd.it (M.C.D.L.); giulia.zardinoni@unipd.it (G.Z.); samathmikaravi@gmail.com (S.R.); squart@unipd.it (A.S.); giuseppe.concheri@unipd.it (G.C.); 2 National Sugar Crop Improvement Centre, College of Advanced Agriculture and Ecological Environment, Heilongjiang University, 74 Xuefu Road, Harbin 150080, China; genggui01@163.com (G.G.); wangyuguang@hlju.edu.cn (Y.W.)

**Keywords:** soil–plant–microbiome interactions, DNA sequencing, multi-omics integration, synthetic microbial communities, sustainable agriculture

## Abstract

Interactions among plant roots, soil, and microorganisms in the rhizosphere regulate nutrient cycling, plant health, and ecosystem resilience. Recent advances in DNA sequencing and multi-omics are contributing to a shift from primarily descriptive surveys toward more mechanistic and predictive frameworks. This review synthesizes methodological developments and conceptual insights spanning microbial ecology, functional genomics, and agricultural applications. We first summarize DNA-based approaches—marker-gene sequencing, shotgun metagenomics, and quantitative nucleic acid assays—and then complementary omics layers, including metatranscriptomics, metaproteomics, metabolomics, epigenomics, ionomics, and phenomics. We next outline computational advances in data integration, network modeling, and visualization that help represent complex multi-layered datasets as biologically interpretable systems. Applications relevant to climate resilience and sustainable agriculture are discussed, including the design of synthetic microbial communities, the identification of biomarkers for soil health and stress tolerance, and case studies in which rhizosphere multi-omics informs crop breeding and soil management strategies. Overall, these developments underscore the potential of treating microbes as functional and, to some extent, manageable components of the plant holobiont. Looking ahead, we identify key research gaps involving standardized workflows, cross-scale causal inference, and real-time monitoring pipelines that integrate molecular diagnostics with remote sensing and edge–cloud analytics. By linking ecological mechanisms with translational practice, multi-omics frameworks may support the development of more sustainable, data-driven agriculture that better aligns productivity with environmental stewardship.

## 1. Introduction

### 1.1. Importance of Soil–Plant–Microbial Communities

Soil–plant–microbial communities constitute a fundamental triad that underpins ecosystem functioning, stability, and productivity. Within the root–soil continuum, interactions across the rhizosphere and the endosphere mediate dynamic exchanges of nutrient fluxes, chemical signaling, and immune regulation. Through this network, plants modulate nutrient acquisition, maintain physiological homeostasis, and adapt to environmental perturbations, thereby shaping plant–microbe community structure and sustaining long-term ecosystem functioning.

Recent studies show growing evidence that beneficial microbial taxa—such as arbuscular mycorrhizal fungi (AMF), nitrogen-fixing bacteria, and plant growth-promoting rhizobacteria (PGPR)—enhance mineral-nutrient uptake, activate stress-responsive signaling pathways, and promote plant performance and resilience [1,2,3]. These interactions are increasingly recognized as core ecological processes that drive plant adaptation and ecosystem resilience.

Microorganisms play a central role in sustaining biogeochemical cycles and ecosystem functioning. By mediating organic matter decomposition, nitrogen and phosphorus mineralization, and nutrient redistribution, soil microbes determine the bioavailability of key elements, influencing the limits of terrestrial primary productivity [2,3,4]. Thus, variation in the composition and activity of microbial functional guilds strongly influences ecosystem carbon sequestration potential and buffering capacity against environmental fluctuations [5].

In addition, plant–soil feedbacks (PSFs) have been widely recognized as fundamental mechanisms shaping species diversity and community dynamics. Plants can modify the composition and function of soil microbial assemblages, generating positive or negative feedback on conspecific offspring performance and thereby influencing interspecific competitive outcomes [6,7]. Recent studies suggest that microbially mediated feedbacks may strengthen niche complementarity among coexisting species and act as key intermediaries in sustaining ecosystem multifunctionality [8].

Amid accelerating global environmental change, microbial community responses have emerged as critical regulators of ecosystem stability. Climatic factors such as elevated temperatures, drought, and altered CO_2_ concentrations can restructure microbial communities and rewire plant–microbe interaction pathways, ultimately reshaping plant adaptive trajectories and functional outcomes [2,3,4]. Structurally robust microbial consortia are thought to enhance system-level functional redundancy and response diversity, thereby buffering ecosystems against environmental shocks [5].

Finally, soil microbiomes not only modulate individual plant traits but also influence community-level outcomes by regulating the balance between niche complementarity and selection effects. This microbial mediation drives the coevolution of plant community composition, productivity, and functional stability [6,8], providing a mechanistic bridge between biodiversity and ecosystem functioning.

To dissect these complex and multiscale interactions, recent advances in DNA-based and multi-omics technologies have become indispensable tools for characterizing microbial community composition, functional potential, and activity across soil–plant compartments. In the following sections, we first outline the ecological and spatial organization of plant-associated microbiomes and then critically review current molecular and omics-based approaches used to interrogate these systems, before discussing integrative frameworks and emerging technologies that promise to move the field toward predictive and mechanistic understanding.

### 1.2. Microbial Roles in Plant Health, Growth, and Stress Resilience

Plant-associated microbial communities play essential roles in regulating host physiological functions and maintaining ecosystem stability. As integral members of the plant holobiont, these communities mediate complex interactions between plants and their environment across the rhizosphere, phyllosphere, and endosphere. Microbes modulate nutrient acquisition, phytohormone homeostasis, stress-responsive signaling, and immune responses, ultimately shaping plant development, phenotypic plasticity, and adaptive capacity [9,10]. Given their multifaceted regulatory roles, there is a growing imperative to leverage DNA sequencing and complementary multi-omics approaches to resolve the composition, functional potential, activity, and temporal dynamics of these microbial assemblages. Certain microbial taxa—including AMF and PGPR—facilitate plant acquisition of essential nutrients through biological nitrogen fixation (by diazotrophic bacteria), phosphate solubilization, siderophore-mediated metal acquisition, and mycorrhiza-mediated phosphorus acquisition via extraradical hyphae. These microbes synthesize key phytohormones—such as indole-3-acetic acid (IAA) and gibberellins—and produce ACC deaminase, an enzyme that lowers plant ethylene (ET) levels by deaminating its precursor, 1-aminocyclopropane-1-carboxylic acid (ACC), thereby influencing root architecture and enhancing shoot biomass [11,12]. These traits are particularly valuable under nutrient-deficient and fluctuating environmental conditions. Recent metagenomic and metabolomic studies have identified numerous microbial genes and pathways involved in nutrient cycling and phytohormone modulation [13].

Microbial consortia in the phyllosphere and the seed endosphere make substantive contributions to plant growth, disease resistance, and abiotic stress tolerance. Leaf-associated microbes contribute to systemic immune priming, improving overall stress tolerance. Seed endophytes undergo vertical transmission to offspring, shaping early microbiome assembly and preconditioning seedlings to withstand environmental challenges [14,15]. Organ-specific microbiomes constitute a continuous, developmentally dynamic microbial interface whose spatiotemporal composition is increasingly resolved by spatial transcriptomics and tissue-specific profiling technologies [10].

Under abiotic stress conditions such as drought, salinity, or heavy metal exposure, stress-responsive microbiomes contribute to host tolerance through modulation of reactive oxygen species (ROS) scavenging, osmotic regulation, and expression of stress-related genes [16,17]. Specific rhizosphere microbes activate key plant hormonal pathways, including abscisic acid (ABA) and jasmonic acid (JA), and produce osmoprotectants and volatile organic compounds (VOCs) that mitigate cellular damage. Notably, microbial signals have been shown to trigger host epigenetic modifications—including DNA methylation and histone alterations—that drive rapid and heritable phenotypic adaptations [18]. Integrated multi-omics platforms combining epigenomics, transcriptomics, and metabolomics are beginning to elucidate the molecular underpinnings of such host–microbe interactions.

With advances in the engineering of synthetic microbial communities (SynComs) and in targeted selection, the plant microbiome is increasingly regarded as a manipulable trait. Experimental evolution studies have shown that enrichment of specific microbial consortia can reproducibly modulate key plant phenotypes—such as flowering time and biomass—across plant genotypes [19]. Emerging concepts such as “keystone taxa” and defense-associated microbiomes (“defense biome”) are used to describe microbial groups that exert a disproportionate influence on host stress resilience and phenotypic outcomes relative to their abundance. Guided by meta-omics analyses, researchers are now assembling functional microbial consortia for applications in precision agriculture and microbiome-based trait engineering [20]. Overall, plant-associated microbial communities function as a central regulatory interface that governs plant health, productivity, and stress resilience. Their interactions with the host involve multifaceted nutrient, hormonal, and signaling networks that are only beginning to be understood. Ongoing advances in high-throughput sequencing and multi-omics integration are clarifying these functional connections, paving the way for a deeper discussion of microbiome-mediated adaptation and its applications in sustainable agriculture.

### 1.3. From Classical Microbiology to Multi-Omics Technologies

Historically, the study of microorganisms has relied predominantly on axenic cultures and phenotype-based assays. However, cultivation-based approaches capture only a small fraction of natural diversity. A global analysis of amplicon, metagenomic, and metatranscriptomic datasets estimated that approximately 81% of microbial cells in major nonhuman environments belong to uncultured genera or higher taxonomic ranks, underscoring how this large uncultured majority limits our ability to characterize microbial diversity, community structure, and ecological functions [21].

The advent of metagenomics marked a paradigm shift by enabling taxonomic and functional profiling of entire microbial communities without the need for cultivation. Subsequently, metatranscriptomics, metaproteomics, and metabolomics extended these insights by revealing what microbes express, what proteins they synthesize, and what metabolites they produce under specific environmental or host-associated conditions [22,23]. Collectively, these omics layers form the foundation of modern microbial systems ecology.

Functional inference tools such as Phylogenetic Investigation of Communities by Reconstruction of Unobserved States 2 (PICRUSt2) [24] and Functional Annotation of Prokaryotic Taxa (FAPROTAX) [25] have further extended the utility of 16S ribosomal RNA (16S rRNA) gene sequencing by enabling phylogenetically informed predictions of microbial metabolic pathways and ecological functions across diverse environments. Concurrently, Knight et al. [26] established best-practice pipelines spanning sampling, DNA extraction, sequencing, and statistical analysis to enhance reproducibility and ensure data comparability across microbiome studies.

In parallel, single-cell omics technologies, including single-cell RNA sequencing (scRNA-seq) and integrated single-cell multi-omics, have enabled high-resolution profiling of microbial heterogeneity, thereby shifting the focus from population-averaged signals to cell-specific functional landscapes [27]. These approaches have uncovered rare biosphere members and revealed niche-specific metabolic functions within rhizosphere systems.

The integration of multi-omics data has become a central challenge, limited by data heterogeneity, normalization inconsistencies, and temporal misalignment [28]. To address these issues, emerging approaches utilize machine learning and network modeling to map inter-layer dependencies and predict system-level behavior. Importantly, understanding system-level interactions also requires spatial resolution, identifying not only which molecular components are involved but also in which cell types and tissue contexts these interactions occur. Incorporating such localization data can greatly enhance the interpretability of multi-omics networks and provide mechanistic insight into soil–plant–microbe system dynamics.

Moreover, omics applications in soil environments are challenged by the complexity of matrices, including non-target DNA contamination and humic acid interference. Differential lysis, target enrichment, and physical separation protocols have been proposed to enhance DNA recovery and downstream interpretability [23,29]. Although initially applied in biomedical contexts [30], the strategies for omics integration and network inference are broadly transferable to understanding complex interactions within soil–plant–microbe systems. Continued advances in omics technologies are now redefining the analytical boundaries of microbial ecology, enabling quantitative predictions of how soil–plant–microbe networks respond to environmental change.

### 1.4. Objectives and Scope of the Review

Soil–plant–microbial communities constitute a critical interface that governs plant productivity, nutrient cycling, stress adaptation, and ecosystem resilience. Yet, our understanding of their functional dynamics remains fragmented. Although methods are advancing rapidly, they are still insufficiently integrated, and the translational pathway from ecological insight to agricultural practice remains underdeveloped.

To bridge these gaps, this review adopts integrative multi-omics as a unifying framework and develops a cross-scale synthesis linking mechanistic explanation, molecular and functional profiling, systems-level data integration, and translational applications. We first outline the principal ecological niches and interaction patterns within soil–plant–microbe systems, followed by a discussion of key methodological platforms for DNA extraction, marker-gene sequencing, metagenomics, and quantitative nucleic-acid analysis. Subsequent sections integrate recent advances in metatranscriptomics, metaproteomics, metabolomics, epigenomics, ionomics, and phenomics, together with computational strategies for data integration, modeling, and network inference. Finally, we explore how these technological and analytical developments are shaping applied research on climate resilience and sustainable agriculture, with particular attention to key priorities for standardization, cross-scale causal validation, and real-time monitoring of microbial dynamics.

Building on this framework, the review concludes with an integrated outlook that connects fundamental multi-omics discoveries with practical pathways for microbiome-informed agricultural innovation.

## 2. Overview of Soil–Plant–Microbial Interactions

### 2.1. Rhizosphere, Endosphere, and Phyllosphere Microbiomes

The plant microbiome consists of microbial communities that inhabit distinct ecological compartments of the plant—the rhizosphere, endosphere, and phyllosphere. The rhizosphere refers to the narrow zone of soil directly influenced by root exudates, where a nutrient-rich microenvironment supports a dense and dynamic microbial assemblage. The endosphere encompasses microorganisms residing within plant tissues, including roots, stems, and leaves, without eliciting pathogenic effects. In contrast, the phyllosphere includes all aboveground plant surfaces inhabited by microbes that are exposed to light, desiccation, and variable thermal regimes [10].

Each of these habitats supports distinct microbial communities influenced by plant genotype, root exudate composition, and surrounding environmental factors, notably soil physicochemical properties. It has been demonstrated through metagenomic profiling of calamint (*Satureja nepeta*) rhizospheres that soil pH and organic matter content explain most of the variation in microbial community structure [31]. This finding underscores the dominant role of soil characteristics in shaping the plant microbiome, supporting the model of the “plant–microbe–soil triangle”.

Core microbial taxa, including *Pseudomonas*, *Streptomyces*, and *Burkholderia*, frequently occur across multiple plant species and ecological compartments. These microorganisms commonly participate in nutrient mobilization, antimicrobial defense, and phytohormone regulation [32]. Furthermore, the spatial and temporal dynamics of the plant microbiome are highly responsive to seasonality, developmental stage, and agricultural management practices [33].

These ecological contrasts translate into compartment-specific methodological constraints for molecular profiling [33]. Rhizosphere samples are affected by soil-derived inhibitors and DNA adsorption [31,33], whereas endosphere analyses are often biased by host DNA contamination [32]. By contrast, the low microbial biomass of the phyllosphere makes extraction efficiency and contamination control particularly critical [33].

SynComs are increasingly employed to investigate and engineer plant microbiomes. Tariq et al. [34] reported the use of SynComs comprising stress-tolerant rhizobacteria that enhanced wheat resistance to Fusarium wilt and improved water-use efficiency, illustrating the potential of microbiome-based approaches in enhancing crop resilience.

Despite differing habitats and stress exposure, the rhizosphere, endosphere, and phyllosphere microbiomes form a coherent functional network that supports plant health, nutrient uptake, and stress tolerance across changing environmental conditions.

### 2.2. Symbiotic vs. Pathogenic Relationships

Plant-associated microorganisms engage in a continuum of interactions that span from mutualism to pathogenicity. These relationships encompass a broad ecological spectrum in which microbial behavior and outcomes depend on host genotype, environmental context, and community composition. At the beneficial end of this continuum, mutualistic microbes contribute to nutrient exchange, stress mitigation, and signaling balance, whereas pathogenic organisms disrupt host metabolism and trigger immune defenses. Classic examples include *Rhizobium* and AMF, which form nodules and arbuscules that mediate nutrient symbiosis, contrasted with opportunistic or pathogenic taxa that exploit host resources under stress conditions. Such interaction plasticity highlights the dynamic nature of the plant holobiont, where symbiosis and pathogenicity often represent context-dependent outcomes of the same ecological framework.

Conversely, pathogenic microorganisms such as *Fusarium oxysporum*, *Ralstonia solanacearum*, and *Pythium ultimum* undermine plant health by secreting cell-wall-degrading enzymes and virulence factors and by overcoming or evading plant immune responses, which together lead to wilt, necrosis, or systemic infections.

The equilibrium between symbiotic and pathogenic interactions is tightly modulated by the plant immune system, which integrates both local and systemic defense responses.

Systemic acquired resistance (SAR) and induced systemic resistance (ISR) represent two major defense pathways in plants. SAR is typically elicited by necrotizing pathogens and is mediated through salicylic acid (SA) signaling, whereas ISR is activated by beneficial rhizobacteria and mediated via jasmonic acid (JA) and ET pathways. Chen et al. [32] demonstrated that VOCs emitted by *Bacillus velezensis* can prime ISR in *Arabidopsis*, thereby markedly reducing *Pseudomonas syringae* infection. Tang et al. [35] emphasized the dual functionality of VOCs as both antimicrobial agents and immune activators within rhizosphere defense networks.

Microbial signals not only shape the structure of the plant microbiome but also serve as molecular triggers for host immunity, reinforcing the integrative concept of the microbiome as an extension of the plant immune system.

### 2.3. Microbiome Functions in Nutrient Cycling, Disease Suppression, and Stress Tolerance

The plant microbiome plays a pivotal role in mediating biogeochemical processes, particularly those involved in nutrient mobilization, including nitrogen (N), phosphorus (P), zinc (Zn), silicon (Si), potassium (K), and iron (Fe). Nitrogen-fixing bacteria such as *Azotobacter* and *Rhizobium* contribute to atmospheric nitrogen fixation, whereas phosphate-solubilizing bacteria, including *Bacillus* and *Pseudomonas,* transform insoluble phosphate compounds into plant-available forms. For example, Ansabayeva et al. [36] summarized that inoculation with phosphate-solubilizing PGPR enhances phosphorus uptake under nutrient-deficient conditions, thereby supporting their application in sustainable fertilization strategies.

In terms of disease suppression, beneficial microorganisms inhibit pathogens through multiple mechanisms, including competition for space and nutrients, production of antimicrobial compounds, and interference with quorum sensing [37]. Moreover, these microbes can activate host immune responses—such as ISR—and contribute to the development of soil suppressiveness. At the community level, SynComs integrating multiple beneficial taxa have shown multifunctional potential, simultaneously contributing to pathogen suppression and abiotic stress mitigation [34]. Such microbial interactions also contribute to improved abiotic stress resilience by regulating plant metabolism, enhancing antioxidant enzyme activities such as superoxide dismutase (SOD) and peroxidase (POD), and facilitating osmolyte accumulation. This is exemplified by drought-adapted endophytic bacteria, which further reinforce antioxidant defenses and osmotic regulation, leading to improved drought tolerance [33].

The cumulative effects of microbial-mediated nutrient acquisition, immune regulation, and stress tolerance highlight the microbiome’s potential as a key target for engineering resilient and sustainable cropping systems.

## 3. DNA-Based Methods for Microbial Profiling

### 3.1. DNA Extraction Challenges in Soil and Plant Tissues

Understanding microbial communities within soil–plant systems relies heavily on reliable molecular methods. Among these, DNA-based profiling techniques are indispensable for characterizing the composition, diversity, and potential functional attributes of microbial consortia across diverse niches.

Efficient DNA extraction is a critical prerequisite for any molecular microbiome study. However, soil and plant tissues pose unique challenges due to their complex physicochemical properties and the presence of polymerase chain reaction (PCR) inhibitors such as humic substances, polyphenols, and polysaccharides. These compounds often co-purify with nucleic acids and interfere with downstream enzymatic reactions, thereby reducing amplification efficiency and accuracy [38,39].

As soil surveys expand to larger spatial and temporal scales, the efficiency and throughput of DNA extraction have become major bottlenecks. Recently, automated magnetic bead–based workflows have been evaluated and shown to yield soil DNA with purity and qPCR amplifiability comparable to widely used commercial kits, while substantially reducing hands-on time, processing costs, and operator-dependent variability [40]. Furthermore, differential cell wall structures, as well as variations in physiological states, among microbial taxa (e.g., Gram-positive bacteria, fungi, and archaea) can introduce extraction biases, leading to the underrepresentation of certain lineages. Standardized commercial kits, such as the DNeasy PowerSoil kit, are widely used; however, studies have shown that they may not completely lyse all microbial cells, particularly actinobacteria and endospores [39]. Key features of commonly used commercial soil DNA extraction kits—including their lysis methods, sample types, and typical downstream applications—are compiled in a comparative overview (Table 1) [39,40].

In addition, environmental DNA can originate from both living and dead organisms. The inclusion of relic DNA derived from lysed or inactive cells can bias interpretations of active microbial populations, thereby confounding ecological inferences [38]. Therefore, DNA extraction protocols should be optimized according to sample type and validated through the use of internal controls and mock communities whenever possible [41].

### 3.2. Marker Gene Approaches (16S rRNA, ITS)

Marker gene sequencing, which targets genes that are conserved within a taxonomic group but contain variable regions allowing discrimination between taxa (e.g., the 16S rRNA gene for bacteria and the ITS region for fungi), is a cornerstone technique in microbial ecology, particularly for profiling bacterial and fungal communities. The 16S rRNA gene, used primarily for prokaryotic taxa, and the internal transcribed spacer (ITS) region for fungi—including the ITS1 and ITS2 subregions—provide cost-effective and scalable means to assess community composition and diversity. Recent advances in high-resolution bioinformatic pipelines, such as Divisive Amplicon Denoising Algorithm 2 (DADA2), have enabled the identification of amplicon sequence variants (ASVs), which in turn improve taxonomic resolution and species-level accuracy [42].

However, primer selection and amplification biases remain critical concerns. Conventional fungal primers may underrepresent certain clades due to length and sequence mismatches, which has prompted the development of alternative primer sets like fITS7 and gITS7 for ITS2 analysis [43].

The lack of a unified “best practice” for amplicon-based studies poses significant challenges to reproducibility [41]. Consequently, consensus standards for marker region selection, sequencing platform, and data processing are essential to ensure comparability between studies.

### 3.3. Shotgun Metagenomics

Shotgun metagenomics enables a comprehensive characterization of microbial communities by sequencing all DNA present in a sample without prior amplification. Unlike marker gene approaches (e.g., 16S rRNA or ITS), this method provides both taxonomic and functional resolution, allowing for the reconstruction of genomes, metabolic pathways, and antimicrobial resistance gene profiles from complex microbiomes [44].

**Figure 1:**
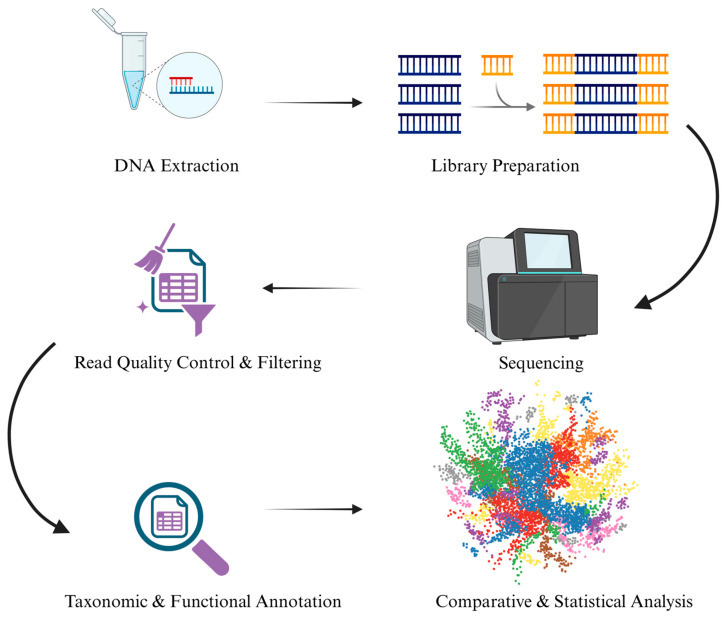
Non-targeted metagenomics analysis workflow.

A typical shotgun metagenomic workflow includes DNA extraction, fragmentation, library preparation, and high-throughput sequencing (e.g., Illumina or Oxford Nanopore), followed by quality filtering, host genome removal, taxonomic profiling, and functional annotation. These analytical steps require substantial computational resources and meticulous bioinformatic design (Figure 1).

Shotgun metagenomics is particularly valuable in soil and plant microbiome research, where microbial diversity is exceptionally high, and many taxa lack representative reference genomes. A recent large-scale effort by Almeida et al. [45] constructed a catalog of 204,938 reference genomes from the human gut microbiome, which serves as a model for enhancing genome recovery and environmental annotation pipelines.

### 3.4. Bioinformatics Pipelines and Databases

As microbial sequencing datasets continue to grow in scale and complexity, the need for robust, reproducible, and scalable bioinformatics pipelines has become increasingly critical. These workflows typically integrate sequence filtering, quality control, taxonomic classification, and functional annotation. The choice of software tools and reference databases directly influences analytical outcomes, making standardization a central objective in microbiome research [26].

**Figure 2:**
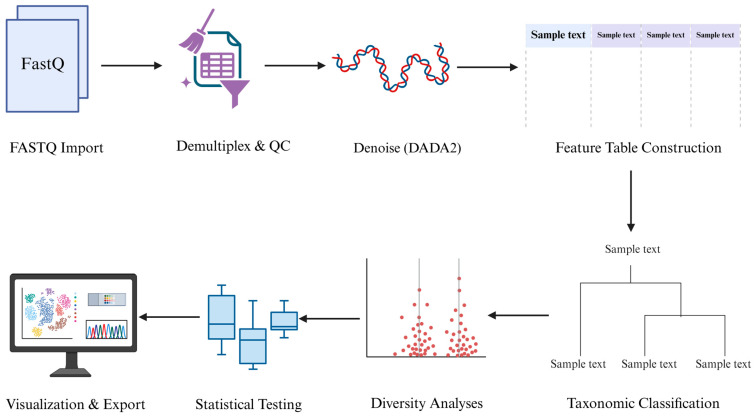
General amplicon sequencing analysis workflow.

For marker gene studies, the most widely adopted platform is Quantitative Insights Into Microbial Ecology 2 (QIIME2). Designed as a plugin-based modular framework, QIIME2 enables seamless integration of denoising (e.g., DADA2), taxonomic classification, diversity analysis, and graphical visualization. It also supports provenance tracking to ensure transparency and reproducibility across all analytical steps [46]. A general amplicon sequencing analysis workflow, covering demultiplexing, quality control, denoising, feature table construction, taxonomic assignment, and downstream diversity analyses, is outlined (Figure 2).

**Figure 3:**
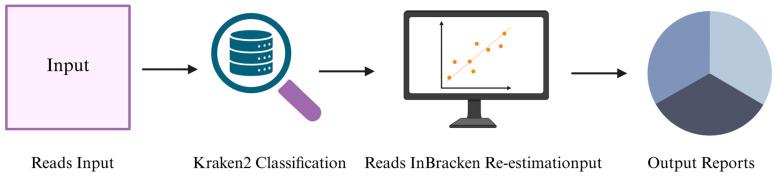
Kraken2 and Bracken classification workflow.

The computational workflow used for read-level taxonomic classification with Kraken2 and Bracken is shown (Figure 3). In the context of metagenomic sequencing, a non-targeted approach, tools such as Kraken2 and Bracken provide efficient read-level taxonomic classification. Kraken2 employs exact *k*-mer matching for ultra-fast taxonomic assignment, while Bracken refines abundance estimates by probabilistically reassigning ambiguous reads to their most likely source taxa [47,48].

A critical component of these workflows is the reference database. For 16S rRNA–based bacterial studies, databases such as the SILVA rRNA gene database (SILVA), Greengenes, and the Ribosomal Database Project (RDP) are most frequently employed. For ITS-based fungal studies, the UNITE database for molecular identification of fungi (UNITE) and RefSeq Targeted Loci (RTL) are commonly used to provide curated and up-to-date fungal reference sequences. In metagenomic contexts, resources such as the National Center for Biotechnology Information (NCBI) Reference Sequence Database (RefSeq), the Genome Taxonomy Database (GTDB), and Metagenomic Phylogenetic Analysis (MetaPhlAn) reference profiles offer broader taxonomic coverage and improved classification consistency. Each database differs in update frequency, phylogenetic depth, and curation quality, which can ultimately influence analytical outcomes and comparability across studies [26].

### 3.5. Quantitative Approaches: qPCR and Digital PCR

While high-throughput sequencing (HTS) provides profound insights into microbial community composition, it remains inherently semi-quantitative, yielding relative rather than absolute abundance estimates. To obtain true quantification, quantitative real-time PCR (qPCR) and digital PCR (dPCR) are widely applied as complementary techniques to sequencing-based approaches [49].

In qPCR, DNA amplification is monitored in real time using fluorescent dyes or sequence-specific probes. The cycle threshold (Ct) value is then used to calculate gene copy numbers based on a standard curve generated from known template concentrations. Although highly sensitive, qPCR can be affected by biases arising from amplification efficiency, primer specificity, and the presence of PCR inhibitors commonly found in soil and plant samples [49].

**Figure 4:**
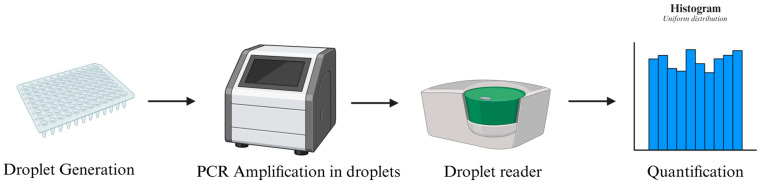
Conceptual workflow of droplet digital PCR (ddPCR).

Digital PCR (dPCR), and more specifically droplet digital PCR (ddPCR), overcomes many of the limitations associated with qPCR by partitioning the reaction mixture into thousands of nanoliter-sized droplets, where PCR amplification occurs independently (Figure 4) [50]. The binary outcome of each droplet (positive or negative) is then used to calculate absolute copy numbers based on Poisson statistics, eliminating the need for external standard curves.

In complex matrices such as soil, where PCR inhibitors, including humic acids, are abundant, dPCR often outperforms qPCR in terms of sensitivity, reproducibility, and dynamic range [51]. Moreover, digital PCR enables multiplex detection and quantification of rare genetic targets, such as antibiotic resistance genes (ARGs), making it particularly valuable for applications in environmental microbiology [52].

To ensure reproducibility and transparency in qPCR-based studies, the Minimum Information for Publication of Quantitative Real-Time PCR Experiments (MIQE) guidelines were established. These guidelines emphasize rigorous primer design, validation of amplification efficiency, inclusion of appropriate positive and negative controls, and comprehensive reporting of experimental metadata [49].

## 4. Advancements in Multi-Omics Techniques

### 4.1. Metatranscriptomics: Revealing Active Microbial Gene Expression in the Rhizosphere

Metatranscriptomics has emerged as a cutting-edge approach to profile the actively transcribed genes of complex microbial communities in situ [53]. In contrast to DNA-based techniques that indicate the potential functional capacity, metatranscriptomics provides insight into real-time microbial activity, thereby offering a powerful means to elucidate dynamic rhizosphere processes [54].

Recent studies have applied this approach to elucidate how beneficial microbes modulate gene expression in response to environmental and plant-derived signals. Under nutrient-limited soil conditions, microbial inoculation markedly upregulates genes involved in siderophore production, auxin biosynthesis, and phosphate solubilization, thereby enhancing plant growth [55]. Notably, siderophore production can also be induced by high levels of other metals, and siderophores may be produced not only by microbes but also by plants, such as in monocots. Similarly, desiccation or drought stress the induction of bacterial osmoprotective genes—including those encoding enzymes for trehalose biosynthesis, glycine betaine accumulation, and transporter proteins—sustaining microbial survival and enhancing plant tolerance [56].

In addition to stress responses, microbial transcriptional dynamics are closely linked to host regulatory processes in the rhizosphere, and plant hormonal and transcriptomic reprogramming under stress provides an important context for interpreting rhizosphere activity [57].

Furthermore, genes involved in nitrogen fixation (*nifH*) and nitrate reduction (*narG*) are actively expressed by free-living and rhizosphere-associated nitrogen-fixing bacteria in response to plant demand, thereby improving nitrogen-use efficiency in maize [58].

These findings illustrate that metatranscriptomics not only catalogues gene activity but also reveals functional responses of microbial communities in relation to plant signals, providing integrated insights across multiple studies.

To further enhance the functional interpretation of transcriptomic data, researchers have begun integrating multi-omics approaches. Noviana et al. [59] provided an overview of rhizosphere meta-transcriptomics within a plant microbiome engineering framework, summarizing methodological considerations and representative applications. In parallel, Tamrakar et al. [60] described optimized RNA extraction coupled with universal ribosomal RNA depletion strategies for assessing rhizosphere microbial activity, strengthening the technical foundation for transcriptomics-based studies.

### 4.2. Metaproteomics: Protein-Level Insights into Community Function

While transcriptomic studies provide valuable insight into the functional potential and regulatory responses of microbial communities, metaproteomics characterizes the actual proteins produced by the microbiota under specific environmental and host-associated conditions [61,62]. By identifying and quantifying proteins expressed in situ, this approach bridges the gap between gene expression and realized biochemical activity.

Extending metatranscriptomic regulation, metaproteomics quantifies the proteins that enact these signals, offering direct evidence of functional realization beyond gene- or transcript-level predictions.

In a rhizosphere case study, Lin et al. [63] applied shotgun metaproteomics to ratoon sugarcane soils and identified proteins associated with nitrogen assimilation, oxidative stress mitigation, and phosphate metabolism. Enzymes such as glutamine synthetase and alkaline phosphatase were highlighted as central to nutrient mobilization and root health, illustrating how microbial protein expression supports plant performance. In a methodological evaluation, Rana et al. [64] compared shotgun proteomics employing label-free quantification (LFQ) with two-dimensional gel electrophoresis (2D-GE) in rhizosphere metaproteomic workflows. They emphasized that accurate taxonomic and functional annotation remains a major challenge due to incomplete reference databases and homology-driven redundancy, particularly in diverse soil microbiomes. From a functional ecology perspective, Chukwuneme et al. [65] reviewed driving forces and monitoring approaches for rhizosphere microbiome diversity and function, and noted that stress-responsive processes and ATP-binding cassette (ABC) transporters are frequently discussed as informative indicators with potential links to plant performance.

These findings highlight metaproteomics as a powerful approach for resolving the biochemical processes underlying microbe–host interactions. By delivering a direct, protein-level view of microbial activity beyond gene- or transcript-level assays, metaproteomics enables a more detailed analysis of molecular crosstalk in rhizosphere ecosystems.

This layer complements metabolomics by linking expressed enzymes to the metabolites they produce, elucidating active pathways invisible to nucleic acid omics alone.

### 4.3. Metabolomics: Microbial Metabolite Interactions with Plants

Metabolomics has become an essential tool for deciphering the chemical language mediating plant–microbiota interactions. In the rhizosphere, where root-secreted metabolites act as both nutrient sources and signaling molecules, metabolomics delineates the biochemical interactions that shape microbial recruitment, community composition, and functional processes, and captures dynamic endpoint fluxes and feedback loops—such as metabolite-induced gene shifts—that uniquely resolve the tangible outcomes of plant-microbe dialogue.

Liu et al. [66] characterized the boron-efficient rhizosphere microbiota and found that specific secondary metabolites in root exudates modulated bacterial gene expression and enhanced plant tolerance to boron deficiency. Their study highlighted the feedback loops between host nutritional status and microbial metabolic pathways.

Root-derived metabolites play a fundamental role in shaping the microbial community. For instance, Wen et al. [67] demonstrated that root-exuded metabolites (including organic acids and other low-molecular-weight compounds) can selectively enrich beneficial microbes (e.g., *Pseudomonas* spp.), contributing to pathogen suppression and improved plant performance. Complementarily, Hafner et al. [68] examined the temporal development of root-exudate profiles and noted that early developmental stages release distinct metabolite signatures that shape microbial succession in a stage-specific manner.

Moreover, Fracchia et al. [69] used metabolomics to characterize dynamic changes in Populus root exudates and associated metabolomes during microbial colonisation, linking temporal variation in exudate chemistry to microbial activity and colonization patterns. Similarly, flavonoids including kaempferol have been shown to act as selective cues shaping root-associated bacterial recruitment and stress-related responses, highlighting metabolite–microbiome co-regulation mechanisms [70].

Metabolomics provides a high-resolution lens on how plants communicate with and influence their microbiomes. Root metabolites act as ecological filters that shape microbial diversity and function, ultimately affecting plant health, stress tolerance, and nutrient-use efficiency. Integrating with upstream omics, metabolomics validates genomic predictions through measured chemical exchanges, offering actionable insights for microbiome engineering that sequence data cannot provide.

### 4.4. Epigenomics and Microbial Influence on Host Gene Regulation

Epigenomics investigates the heritable regulation of gene expression independent of changes in the underlying DNA sequence, encompassing DNA methylation, histone modifications, and non-coding RNA–mediated mechanisms. In plants, accumulating evidence indicates that rhizosphere and endophytic microbes can directly or indirectly reprogram the host epigenome, thereby modulating stress responses, development, and immunity.

Sánchez et al. [71] summarized diverse cases where microbial colonization triggered DNA methylation and demethylation changes in *A. thaliana*, showing that epigenetic reprogramming at defense loci contributes to enhanced immune responsiveness under both biotic and abiotic stress conditions. Complementarily, Furci et al. [72] demonstrated that beneficial interactions could induce genome-wide DNA hypomethylation in *A. thaliana*, particularly at promoter regions of resistance genes, resulting in transcriptional priming and more durable quantitative defense.

Histone modifications are also implicated in microbe-induced chromatin reprogramming. Alvarez et al. [73] summarized multiple cases in which plant-associated microbes altered histone acetylation and methylation patterns and linked these chromatin changes to enhanced tolerance under biotic and abiotic stresses. Additionally, exogenous inputs can reshape root epigenomes. For instance, Annacondia et al. [74] reviewed how environmental stresses trigger dynamic reprogramming of DNA methylation and chromatin states, which can in turn modulate hormonal and developmental trade-offs.

Microbial regulation of plant small-RNA pathways has attracted increasing attention. Bacterial inoculation alters Dicer-like protein (DCL) protein activity and the abundance of small interfering RNAs (siRNAs), indicating that the host RNA-silencing machinery can be reprogrammed by the microbiota to fine-tune gene expression under stress [75].

Epigenetic mechanisms position microbial communities as modulators of host transcriptional programs, forming a multilayered regulatory network. These microbe–host interactions bridge ecological microbiology with functional genomics in plants, providing a mechanistic link from community context to gene regulation.

### 4.5. Ionomics and Nutrient Flux in Plant–Microbe Systems

Ionomics, the high-throughput analysis of elemental composition in biological systems, provides a powerful lens to investigate plant–microbe interactions related to nutrient uptake and distribution. Unlike traditional nutrient assays, ionomics captures system-wide elemental signatures that reflect both physiological states and microbial influence.

Wang et al. [76] used ionomic profiling to assess how soil microbial communities reshape nutrient availability in rhizosphere microzones. Their findings revealed microbial regulation of zinc, iron, and manganese uptake, closely linked to root-zone acidification and transporter gene expression. Kaspari et al. [77] further highlighted sodium as a potential ‘seventh macronutrient’ in ecosystem nutrient cycling, emphasizing that microbial processes can mediate nutrient stoichiometry across trophic levels.

Martos et al. [78] analyzed the rhizobiome and shoot ionome of the metal hyperaccumulator Noccaea brachypetala and reported associations between root-associated microbial community features and distinct elemental accumulation patterns.

Zeng et al. [79] found that specific rhizobacteria modulate nitrate and sulfate assimilation by altering the expression of ion transporter genes in both roots and shoots. Their data further indicate that microbially derived organic acids and redox-active metabolites act as regulators of host ion transport and channel activity. Consistent with this, Harbort et al. [80] reported that root-secreted coumarins interact with rhizosphere microbial communities to enhance iron acquisition in *A. thaliana*, establishing a feedback loop linking root exudates, microbial composition, and host nutrient status. Moreover, the availability of specific nutrients can in turn influence which microbial taxa are able to colonize the rhizosphere and plant tissues, highlighting a bidirectional interplay between elemental composition and microbiome assembly.

Ionomics integrated with microbiome data provides a robust avenue for resolving functional nutrient-flux networks. This integrative framework informs microbe-guided strategies to design cropping systems with improved nutrient-use efficiency.

### 4.6. Phenomics and Interactomics in Host–Microbe Interaction Mapping

Phenomics and interactomics represent emerging frontiers in systems biology aimed at decoding complex host–microbe interactions. While phenomics focuses on large-scale quantification of morphological, physiological, and molecular traits under diverse conditions, interactomics elucidates the molecular networks that mediate host responses to microbial colonization and signaling [81,82,83,84].

Ji et al. [85] laid the foundation for bridging next-generation sequencing with phenotypic prediction, arguing for the importance of genotype–phenotype linkage in the context of host–microbiome studies. Xu et al. [86] further advanced the “holo-omics” concept by integrating transcriptomic, proteomic, metabolomic, and phenomic layers to build predictive models of microbial function and its impacts on plant hosts. Daniel et al. [87] demonstrated that high-throughput phenotyping platforms can detect subtle changes in root architecture, stomatal dynamics, and chlorophyll fluorescence caused by microbial inoculants, which were previously undetectable using conventional assays. Mohapatra et al. [88] explored new genetic models that integrate multi-trait phenotyping with quantitative trait loci (QTLs) associated with microbiota-modulated traits, revealing candidate loci linked to microbial responsiveness. Weßling et al. [89] systematically mapped effector–host protein interactions across fungi, bacteria, and oomycetes, demonstrating that evolutionarily divergent pathogens converge on common *A. thaliana* hub proteins, and thereby reshape immune and developmental pathways.

Phenomics and interactomics provide a comprehensive toolkit for decoding how microbial signals are perceived, transduced, and translated into phenotypic outcomes. Their integration enables fine-grained dissection of complex plant–microbe symbioses and supports crop improvement via precision microbiome manipulation.

## 5. Data Integration and Systems Biology Approaches

### 5.1. Challenges in Multidisciplinary Integration

The rapid advancement of multi-omics technologies—such as genomics, transcriptomics, proteomics, metabolomics, and microbiomics—has greatly enhanced our ability to decode the complex ecological interactions between plants and their rhizosphere microbiota. However, the diverse and multilayered nature of these datasets poses significant challenges for integration and interpretation.

Substantial discrepancies exist among different omics platforms in terms of data generation methods, formats, spatial resolution, and temporal dynamics. For instance, transcriptomic datasets capture rapid gene-expression responses, whereas metabolomic profiles often reflect delayed or near-steady-state metabolic changes. Such temporal and spatial mismatches necessitate careful calibration and normalization prior to integrative analysis [90]. Moreover, the lack of standardized metadata annotations and data-sharing protocols hampers cross-study data reusability and undermines comparability and reproducibility. This challenge has motivated the development of reporting standards and Findable, Accessible, Interoperable and Reusable (FAIR) data principles in multiple omics domains—for example, the Minimum Information About a Microarray Experiment (MIAME) for transcriptomics, the Minimum Information About a Proteomics Experiment (MIAPE) for proteomics, the Minimum Information about a Marker Gene Sequence (MIMARKS) and Minimum Information about any (x) Sequence (MIxS) for microbiome sequencing, and the FAIR Guiding Principles for scientific data [91,92,93,94].

Multi-omics integration is further hindered by technical limitations, including batch effects, missing data, and the accumulation of noise across multiple data layers [95]. Microbiome data, for example, are often highly skewed and exhibit significant α- and β-diversity differences across samples, posing challenges for the application of conventional statistical models [96]. Additionally, certain omics such as proteomics and epigenomics remain underrepresented due to technical throughput and cost constraints, limiting their incorporation into large-scale systems modeling.

At its core, multi-omics integration is an inherently interdisciplinary endeavor that requires expertise in plant biology, bioinformatics, microbial ecology, soil science, and statistical modeling. Terminological differences, conflicting modeling paradigms, and inconsistent data-processing workflows across these fields often impede effective cross-disciplinary collaboration [97]. Although several international initiatives have begun to address standardization, most remain at an early stage of development.

In conclusion, although multi-omics integration holds strong potential to drive systemic advances in rhizosphere research, its practical application remains challenged by the lack of metadata standards, interdisciplinary coordination barriers, and multi-scale data complexity. Future efforts should focus on standardizing integration workflows, constructing high-quality and shareable omics repositories, and developing interactive and interpretable visualization platforms to enhance the accessibility and biological interpretability of systems-level insights in rhizosphere ecology.

### 5.2. Computational Tools and Statistical Models

In rhizosphere systems, the multidimensional and high-throughput characteristics of omics data necessitate the use of robust, scalable computational models for data integration and mechanistic inference. Because biological interconnections across different omics layers are rarely linear, advanced modeling strategies that integrate statistical modeling, machine learning, and causal inference are required.

Classical statistical approaches to multi-omics integration include multi-block partial least squares (PLS), extended principal component analysis (PCA), and joint factor analysis (JFA). Among these approaches, the mixOmics suite provides a versatile framework based on PLS and canonical correlation analysis (CCA), enabling the visualization and interpretation of cross-omics correlation structures [98]. Building on this framework, Data Integration Analysis for Biomarker discovery using Latent cOmponents (DIABLO) has been widely adopted for horizontal integration of multiple omics platforms to identify key variable modules for functional prediction.

Recent advances in unsupervised learning have substantially improved multi-omics integration strategies. For example, Multi-Omics Factor Analysis (MOFA) models low-dimensional latent factors to uncover both shared and omics-specific sources of variation, offering high sensitivity for temporal analyses of rhizosphere dynamics [99]. In addition, automated machine learning (AutoML) approaches have been introduced into multi-omics analyses, where they automate feature selection and model tuning, thereby lowering the barrier to broader adoption by non-specialists [96].

Importantly, model interpretability plays a critical role in rhizosphere microbiome multi-omics studies. Predictive accuracy is no longer the sole focus; there is increasing emphasis on characterizing biological interdependencies and regulatory pathways among variables. To this end, graph neural networks (GNNs) and causal inference frameworks are gaining popularity as tools for enhancing mechanistic understanding. This trend reflects a broader shift from predominantly black-box modeling toward more transparent, biologically grounded frameworks for multi-omics integration, as exemplified by recent work on causal and prior-knowledge-driven multi-omics integration [100].

### 5.3. Network Analysis and Functional Annotation

With the rapid accumulation of multi-omics datasets in rhizosphere systems biology, the construction and analysis of cross-omics functional networks have emerged as powerful strategies for deciphering the mechanisms underlying plant–microbiome interactions. Network analysis transforms complex omics measurements into interpretable biological relationship maps that highlight signaling pathways, regulatory elements, and key interaction nodes.

The most widely used network types include co-expression networks, gene regulatory networks (GRNs), metabolic networks, and host–microbe interaction networks. For example, Weighted Gene Co-expression Network Analysis (WGCNA) is frequently employed to identify gene modules that are correlated with environmental cues or phenotypic traits, which can then be cross-annotated with metabolomic or microbiome profiles [101]. Topological features such as node degree and betweenness centrality are commonly used to pinpoint “functional core genes” or “hub microbes”.

In integrated networks, different omics layers are often represented using hyperedges or bipartite and multiplex structures. For example, transcriptomic and metabolomic data can be jointly used to construct pathway-driven networks that help identify key metabolic bottlenecks and regulatory factors, whereas microbiome data can be used to extend the network to include microbial taxa and functions, thereby linking microbial activity to host gene-expression dynamics [102]. Systems-level functional annotation further relies on databases such as the Kyoto Encyclopedia of Genes and Genomes (KEGG), the MetaCyc metabolic pathway database (MetaCyc), the evolutionary genealogy of genes: Non-supervised Orthologous Groups (eggNOG), and the Plant Transcription Factor Database (PlantTFDB) for pathway mapping and Gene Ontology (GO) enrichment analyses.

Temporal dependence is another key characteristic of rhizosphere signaling networks. Several studies have introduced temporal-network and dynamic-graph models to characterize root-associated responses over time. For example, Yuan et al. [103] constructed a tripartite plant–rhizosphere–metabolite network based on successive pathogen-exposure experiments in *A. thaliana*, revealing that plants reprogram their exudation profiles to reshape the microbiome and establish a “soil memory”. Such integrative approaches provide valuable insights into long-term adaptation and community resilience.

Although network analysis has become a central tool in multi-omics research, its reliability and biological relevance largely depend on input-data quality, node-definition strategies, and the timeliness of reference databases. Future network construction in rhizosphere systems should place greater emphasis on cross-platform standardization and model interpretability, underpinned by experimental validation to improve predictive accuracy.

### 5.4. Visualization Platforms for Omics Integration

In multi-omics studies, the high dimensionality and complexity of datasets render visualization an indispensable component of data interpretation. In rhizosphere research, visual mapping of interactions among genes, metabolites, and microbes not only helps researchers discern system-level patterns but also provides intuitive, interpretable representations for broader audiences, including non-specialists.

Mainstream visualization platforms employ network graphs, chord diagrams, heatmaps, and pathway maps to integrate multi-layer omics data. Among these, Cytoscape 3.10.4 remains one of the most widely used tools because of its extensible plugin ecosystem (e.g., ClueGO, stringApp, MetScape), which supports the construction and functional annotation of gene–metabolite and host–microbe interaction networks. OmicsNet further facilitates multi-omics integration through three-dimensional network rendering and ontology-informed mapping, making it particularly suitable for visualizing complex plant–microbe symbioses.

For metabolic pathway analysis, tools such as iPath3.0 and KEGG Mapper enable interactive tracing of metabolic pathways and associated enzymes, thereby linking metabolomic data to transcriptomic profiles. MetaboAnalyst provides graphical interfaces for PCA, partial least squares discriminant analysis (PLS-DA), pathway enrichment, and network or module detection, supporting joint exploration of metabolomic and phenotypic data [104]. In addition, Shiny-based interactive dashboards are gaining popularity, enabling users to deploy web interfaces for dynamic data exploration and communication of results.

Despite the growing number of available tools, substantial challenges remain for multi-omics visualization, including limited visual resolution and scalability, poor interoperability across data dimensions, and insufficient interactivity [91]. Future developments in visualization for rhizosphere omics should prioritize enhanced interactivity, richer semantic annotation, and seamless cross-platform integration. Moreover, explainable visualization—combining AI-assisted layout or diagram recommendation with expert biological interpretation—will be pivotal for making systems-biology approaches more actionable and interpretable.

## 6. Applications in Climate Resilience and Sustainable Agriculture

### 6.1. Microbiome Engineering and Synthetic Communities

The rhizosphere microbiome serves as a crucial mediator of plant–environment interactions and plays central roles in nutrient cycling, signaling, and stress adaptation. Traditional single-strain inoculations, although often effective under controlled conditions, frequently exhibit instability and unpredictability in complex field environments. This limitation has motivated the development of SynComs, which are rationally designed and assembled from functionally complementary strains to reconstruct or optimize community-level functions, thereby providing more controllable, stable, and predictable growth-promoting effects in agricultural systems.

In recent years, the rapid development of multi-omics technologies has provided a solid theoretical and technical foundation for constructing SynComs. Metagenomics and transcriptomics reveal the existence and expression patterns of core functional genes within microbial communities, while metabolomics and proteomics help to decipher metabolic interactions among strains. These data not only aid in the identification of key growth-promoting strains but also guide community optimization and assembly. At the same time, gene-editing tools such as clustered regularly interspaced short palindromic repeats–CRISPR-associated (CRISPR–Cas) systems and high-throughput culturing methods are increasingly being applied to refine functional traits of target microbes, further enhancing community stability and adaptability. Multi-omics–driven community design significantly improves crop performance under stress conditions [34].

In practical applications, SynComs have demonstrated substantial potential to enhance salt tolerance, drought resistance, and disease suppression. Under saline conditions, combinations of salt-tolerant, growth-promoting strains can regulate osmolyte synthesis and mitigate ion toxicity, thereby enhancing plant salt tolerance [105]. Under drought stress, SynComs can improve root water-use efficiency and increase antioxidant activity, thereby raising crop survival rates. Microbiome engineering has been proposed as a strategic approach to address water scarcity and foster sustainable agriculture [106]. Moreover, SynComs have proven effective in disease management by incorporating antagonistic and competitive strains that suppress soil-borne pathogens while simultaneously inducing plant immune responses, thus reducing reliance on chemical pesticides.

Overall, SynComs represent an innovative strategy for translating laboratory advances into field applications, offering practical solutions for coping with environmental stresses and helping to stabilize crop yields. With rational design and optimization guided by multi-omics, SynComs are increasingly regarded as an important tool in driving the green transformation of agriculture.

### 6.2. Microbial Biomarkers for Plant Stress and Soil Health

When plants are exposed to abiotic stresses such as drought, salinity, heat, or heavy metals, their rhizosphere microbial communities often undergo substantial reconfiguration in composition and function. These community-level characteristics are increasingly recognized as key biomarkers that reflect plant stress responses and soil health. Multi-omics approaches enable researchers to identify core microbial taxa and metabolites that are closely associated with plant resilience, thereby offering novel tools for agricultural monitoring and precision management.

Multi-omics studies have highlighted the indicative value of microbial biomarkers under stress conditions. Optimization of rhizosphere microbiomes has been shown to improve crop water-use efficiency and nutrient uptake, while omics analyses reveal functional microbial modules associated with drought and salinity tolerance [107]. The integration of multi-omics and high-throughput sequencing has greatly advanced the characterization of plant responses to abiotic stress, and specific bacterial taxa (e.g., *Pseudomonas* and *Bacillus*) are often correlated with enhanced antioxidant activity and osmolyte accumulation [108]. Furthermore, certain plant growth-promoting microbes (PGPMs) can serve as reliable indicators across contrasting soil types, predicting soil health status and plant adaptive capacity [109].

In agricultural practice, microbial biomarkers hold considerable promise. For instance, shifts in the abundance of certain functional microbes in soil may serve as early indicators of salinization or drought stress, whereas the accumulation of specific metabolites (e.g., proline, volatile organic compounds) can signal oxidative stress in plants. Integrating such biomarkers into management frameworks can not only facilitate dynamic monitoring of soil health but also support precision fertilization, stress-resilient breeding, and ecological restoration. Nevertheless, substantial challenges remain regarding the consistency and generalizability of biomarkers across regions and cropping systems, which currently limits their standardization and adoption in large-scale agricultural applications.

### 6.3. Case Studies: Climate-Resilient Crops and Soil Management

In recent years, the integration of multi-omics into crop improvement and soil management has opened new avenues for coping with climate change. At the crop level, studies have elucidated how plant–microbe interactions contribute to resilience, thereby informing the development of drought-tolerant, salt-tolerant, and nutrient-efficient crop varieties. At the soil level, analyses of microbial community structure and function have increasingly informed management strategies such as crop rotation, cover cropping, residue return, and microbial inoculation.

For instance, in sorghum, integration of plant biotechnology with beneficial microbial interactions has been shown to enhance adaptation to climate-related stresses. In a case study, synergistic effects between drought-tolerant sorghum varieties and their rhizosphere microbiomes were observed, underscoring the potential of combining genetic improvement with rhizosphere modulation [110]. In broader agricultural systems, the combined use of transcriptomics, metabolomics, and microbiome profiling helps identify key signaling pathways and functional microbial taxa, thereby providing a robust foundation for breeding resilient crops and designing precision management strategies [111]. Furthermore, Dwivedi et al. [111] argued that future progress in rhizosphere research should build on case studies to develop integrated approaches linking crop selection, soil management, and ecological restoration.

Crop resilience and soil management are not isolated research domains but interconnected components of a self-reinforcing system. Mechanistic insights into plant–microbe interactions provided by multi-omics not only guide crop breeding and field management but also help safeguard the long-term stability of agricultural production under global climate change.

### 6.4. Transformative Potential of Agricultural Ecosystems

With rapid advances in multi-omics and microbiome engineering, agricultural ecosystems are entering a critical transitional phase toward low-carbon, green, and sustainable development. The application of rhizosphere multi-omics not only provides a theoretical framework for understanding plant–microbe interactions but also enables the efficient utilization of microbial resources in practice. The transformative potential of these approaches lies in integrating microbial functions, crop improvement, and management strategies to shift production systems from high-input systems heavily reliant on fertilizers and pesticides toward precision-based, ecologically informed, and sustainable models.

Raza et al. [112] argued that designing “climate-smart crops” requires integrated strategies that span genetics, rhizosphere microbiome optimization, and agronomic practices to ensure sustainable production and future food security. Pandey and Saharan [113] further emphasized that the soil microbiome constitutes a strategic resource for boosting yields and advancing sustainability, with the potential to substantially reduce chemical inputs. Meanwhile, Verma et al. [114] introduced the concept of “rhizosphere engineering”, proposing that optimizing microbial community structure and function could simultaneously enhance agricultural productivity and ecosystem services, thereby offering a promising pathway toward concurrent agricultural and ecological sustainability.

Rhizosphere multi-omics and microbiome engineering have transformative potential not only at the crop level but also at the ecosystem scale. By integrating technological innovations with optimized management practices, agricultural systems can more effectively pursue both productivity and environmental goals.

## 7. Future Directions and Research Gaps

### 7.1. Standardization of Multi-Omics Workflows

Standardizing multi-omics workflows aims to close the loop linking experimental data and context to computational analysis and machine-actionable data release, thereby ensuring the comparability, reusability, and portability of research outputs.

To this end, MIxS provides a minimum information framework that harmonizes the semantics of sequence and environmental metadata, thereby reducing metadata heterogeneity across studies [94]. In parallel, the Investigation/Study/Assay (ISA) suite structures experimental design, sample provenance, and technical parameters to enable integration across experiment and data types and to improve traceability [115].

At the execution layer, standardization is supported by workflow management systems, containerization projects, and community-curated pipeline ecosystems. Snakemake encodes complex analyses as auditable, declarative workflows that can be scaled from laptops to high-performance computing clusters [116]. BioContainers provides standardized, versioned container images for commonly used bioinformatics tools, mitigating dependency drift and ensuring consistent runtime environments across platforms [117], while the nf-core ecosystem offers a collection of best-practice Nextflow pipelines that institutionalize portability and reproducibility through shared templates, peer review, continuous integration (CI), and versioned releases [118].

For publication and reuse, Research Object Crate (RO-Crate) packages data, code, workflows, and provenance as machine-actionable research objects, linking archival with re-analysis and long-term usability [119].

Standardization should not be limited to method reproducibility but should also target the portability of scientific conclusions. The LongitUdinal modelling with Partial least squares regression for NEtwork inference (LUPINE) framework for longitudinal microbiome network inference provides end-to-end, fully re-computable pipelines with explicit steps, parameters, and evaluation metrics, thereby enabling robust re-analysis and method substitution across seasons, sites, and treatments [120]. This paradigm is consistent with nf-core and RO-Crate best practices and offers a directly transferable path for causal hypothesis testing and deployment in spatio-temporal rhizosphere multi-omics studies.

**Figure 5:**
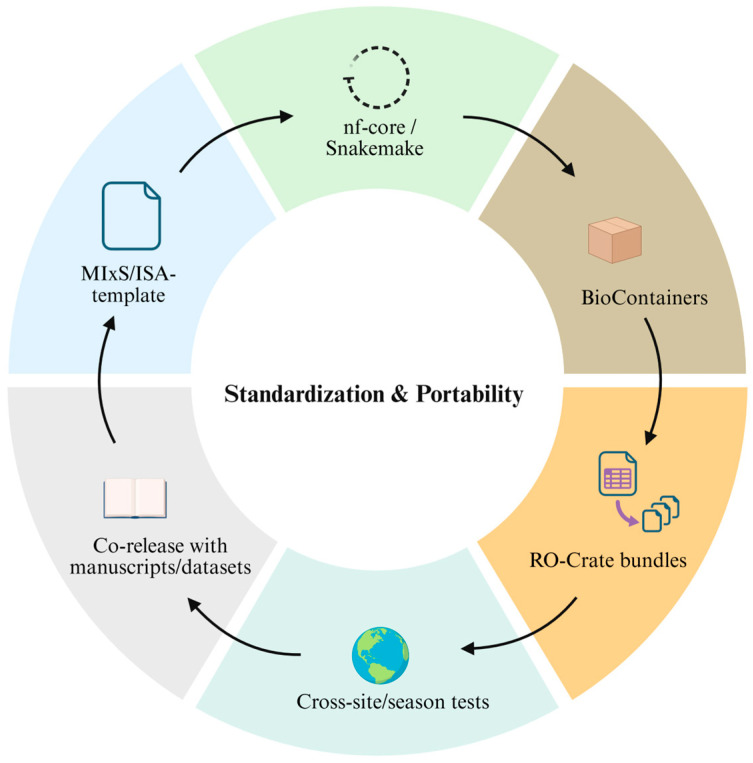
Key Elements of Workflow Standardization and Portability.

Accordingly, we suggest six actionable components to enhance workflow standardization and portability: (i) a MIxS/ISA-based minimal template; (ii) nf-core or Snakemake scaffolds with pinned versions/dependencies; (iii) BioContainers-backed environments; (iv) RO-Crate bundles with persistent identifiers; (v) cross-site/season generalization tests; and (vi) co-release of workflow artefacts with manuscripts and datasets (Figure 5).

### 7.2. Linking Microbiome Data to Plant Phenotypes

The central challenge in linking plant phenotypes with microbiome data is to establish a verifiable, generalizable, and actionable chain of evidence along the path “host genetics → microbiome → environment → trait”.

Multiple lines of evidence now converge on this objective. Large-scale field studies first demonstrated heritable microbial taxa in the rhizosphere, opening the door to quantitative genetics and selection schemes that explicitly consider host–microbe associations [121,122]. Genome-wide mapping then identified host loci that influence community composition, consistent with mechanisms involving root exudation, immunity, and nutrient transport [123]. At a mechanistic level, integrative analyses of host genetics × colonizing bacteria × metabolism provide an explicit bridge from genes to metabolites and community shifts [124]. Comprehensive overviews of host genetic control of plant microbiomes organize general principles and contingencies across crops, tissues, and environments [125,126]. On the “microbiome → trait” side, robust associations have been repeatedly observed linking community diversity and keystone taxa to plant performance, offering interpretable features for prediction and intervention [121,122,125,126].

Methodologically, progress hinges on three dimensions: cross-sectional association, longitudinal inference of causality, and cross-context portability. In cross-sectional designs, variance partitioning and heritability analyses quantify the contributions of host, environment, and microbiome, and test additive gains in genome–microbiome–environment joint models [121,122,123,126]. For causal inference, longitudinal and perturbation designs are essential. LUPINE provides a transparent, open implementation for longitudinal network inference, enabling robust modeling of inter-taxon interactions and lagged effects [120]. Trait-based dynamical models further integrate species traits with ecological processes to predict community trajectories and functions, thereby bringing mechanism-to-phenotype mapping into the time domain [127]. Finally, studies on microbiome-enabled genomic prediction and selection indicate that incorporating microbiome features can enhance predictive accuracy and robustness for multiple traits, creating an avenue for early selection of environmentally sensitive traits.

### 7.3. Real-Time Monitoring and Prospects for Precision Agriculture

The near-term vision for real-time monitoring is to close a field-scale loop that couples rapid typing (portable Nanopore sequencing and CRISPR-based diagnostics) with multi-source sensing—unmanned aerial vehicle (UAV)-based hyperspectral and thermal imaging, in-field probes—and edge–cloud analytics, thereby turning minute-to-hour–scale signals from the rhizosphere–canopy continuum into prescriptive management actions.

For rapid typing, portable Nanopore platforms have demonstrated end-to-end, field-deployable molecular diagnostic workflows—from extraction to identification—in resource-limited settings (e.g., Tree Lab) [128]. The combination of long reads and short turnaround times makes Nanopore sequencing attractive for emergency surveillance and variant tracking, but substantial challenges remain for low-abundance targets and complex matrices, as well as for error control and protocol standardization [129,130,131]. CRISPR–Cas diagnostics (e.g., Cas13a) enable minute-scale nucleic acid detection without deep sequencing and are particularly suitable for RNA viruses and on-farm screening. Together with sequencing, they form a complementary pair—sequencing providing breadth and provenance, CRISPR providing speed and targeted assays [132,133].

For crop state sensing and spatial modeling, UAV-borne hyperspectral and thermal imaging systems can reliably retrieve key crop biophysical variables, including leaf area index (LAI), chlorophyll and nitrogen status, and canopy temperature. Recent reviews and best-practice papers have consolidated procedures for radiometric calibration, ground-truth data collection, acquisition timing, and flight altitude, enabling early stress detection and variable-rate management in precision agriculture [134,135,136]. A comprehensive review of hyperspectral systems for precision agriculture further reports steady gains in sensitivity to water, nutrient, and pest stresses over the period 2003–2023, yet extrapolation across crops and environments still requires rigorous cross-validation and multi-season, multi-site data fusion [135].

**Figure 6:**
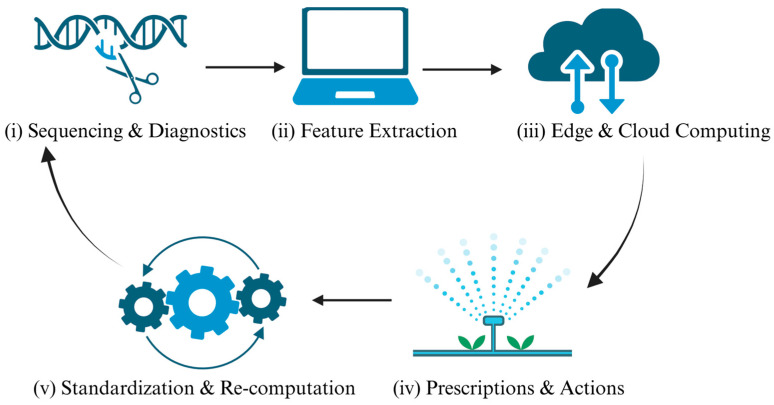
Closed-loop pipeline for multi-omics integration in precision agriculture.

A closed-loop pipeline for precision-agriculture multi-omics integrates (i) sequencing, diagnostics, and sensing (CRISPR-based assays, UAV-borne hyperspectral and thermal imaging, and ground sensors); (ii) feature extraction (pathogen guilds, CRISPR readouts, unmanned aerial vehicle–hyperspectral imaging (UAV-HSI) indices, soil electrical conductivity, and water potential); (iii) edge and cloud computing (lightweight models at the edge and uncertainty quantification in the cloud); (iv) prescriptions and actions (variable-rate irrigation and fertilization, biocontrol, microbial inoculants); and (v) standardization and re-computation (metadata, containerized workflows, and multi-site substitution) (Figure 6) [120,128,129,130,131,132,133,134,135,136].

Challenges and gaps include: limitations in signal-to-noise ratios and quantification for low-copy-number analytes in complex matrices, which require robust extraction protocols, internal controls, and cross-platform benchmarking [128,129,130,131,133]; a lack of standards for multi-source data fusion, including temporal alignment and calibration of UAV-based hyperspectral and thermal imaging with molecular assays [134,135,136]; limited portability under genotype × environment × management interactions, calling for leave-one-site or leave-one-season validation strategies and transfer-learning approaches [135,136]; and cost–benefit and operational constraints, whereby maintenance, training, and consumables must be linked to quantified gains in yield, water savings, and pesticide reduction.

Moreover, deploying such advanced integrated systems globally confronts substantial barriers, including high capital costs for equipment, specialized expertise requirements, and critical infrastructure needs (power, connectivity, cold storage), which remain largely inaccessible to smallholder farmers, necessitating parallel development of low-cost, low-tech alternatives for equitable scalability.

Overall, the speed of sequencing and CRISPR-based assays, the spatial coverage of UAV-based hyperspectral and thermal imaging, and the timeliness of edge–cloud analytics constitute a core capability stack for next-generation precision agriculture.

## 8. Conclusions

Soil–plant–microbe interactions form the foundation of plant performance, resource-use efficiency, and ecosystem stability. Microbial communities inhabiting the rhizosphere, endosphere, and phyllosphere regulate nutrient cycling, immune activation, and tolerance to environmental stresses. Advances in DNA-based methods and multi-omics technologies have shifted research from static descriptions toward functional and dynamic insights. Marker-gene sequencing, metagenomics, and quantitative approaches provide detailed community profiles, whereas transcriptomics, proteomics, metabolomics, and ionomics reveal the active processes that shape host–microbe relationships. These tools collectively enable the identification of keystone taxa, functional pathways, and host–microbe feedbacks, and they support the construction of predictive models that link microbial variation to plant traits and bridge ecological mechanisms with agricultural applications.

Multi-omics has transformed plant–microbe research by linking microbial diversity with functional expression patterns and host responses. At the mechanistic level, it connects community shifts with changes in gene expression, metabolite fluxes, and regulatory networks, uncovering pathways that influence plant growth, immunity, and stress adaptation. On the applied side, multi-omics guides the design of synthetic microbial communities that deliver more consistent benefits under challenging environments and enables the discovery of biomarkers that serve as early indicators of soil health and plant performance. At the conceptual level, integrating multiple omics layers supports a holistic view in which microbes are recognized as active and manageable components of the plant holobiont, thereby laying the groundwork for precision breeding and sustainable, microbiome-informed agriculture.

Turning multi-omics insights into benefits that withstand field testing will require research and application to operate as a continuous pipeline. End-to-end, auditable standards—from sampling through analysis—must harmonize laboratory protocols, metadata, and computational workflows so that results are comparable and reusable. Evidence needs to advance from correlation to causation by linking host genetics, root exudation and defense, microbial interaction networks, and metabolite or ion dynamics to plant traits through longitudinal and perturbation designs, complemented by spatially resolved and single-cell readouts. In practice, rapid diagnostics should be fused with proximal and remote sensing and edge–cloud analytics so that molecular and canopy-level signals become early-warning indicators that trigger targeted, variable-rate management actions. Robust lab-to-field translation pathways are needed to test and harden synthetic consortia and biomarker panels across genotypes, soils, climates, and management regimes, while addressing quality control, biosafety, delivery, regulatory readiness, farmer usability, and cost. External validity will grow with multi-site, multi-season trials that rely on interpretable models, report uncertainty, share benchmarks and effect-size metrics, and publish negative results.

Ultimately, host–microbiome co-design—integrating microbiome features into breeding targets and tuning exudate chemistry and immune set-points, with ionomics and phenomics as readouts—offers a way to align basic mechanisms with breeding and management. These steps will enable soils, plants, and microbes to be managed as a single, data-driven system, thereby turning multi-omics insight into durable and scalable gains in productivity, resilience, and sustainability.

## Figures and Tables

**Table 1:** Comparison of commercial soil DNA extraction kits.

Kit	Sample Amount	Lysis Method	Suitable Samples	Targets	Applications
MoBio PowerSoil^®^	0.25 g	Bead-beating	Soil, fecal, stool, biosolid	Bacteria, fungi, algae, actinomycetes, nematodes	PCR/qPCR/NGS
E.Z.N.A.^®^ Soil DNA	1.00 g	Bead-beating	Clay, sandy, peaty, chalky, loamy soils	Bacteria, fungi, yeast, algae	PCR/Southern blot/NGS
Qiagen DNeasy PowerSoil Pro	0.25 g	Bead tube + chemical	Compost, clay, topsoil	Bacteria, fungi	PCR/qPCR/NGS
MP Biomedicals FastDNA Spin	0.50 g	Bead-beating + chemical	Soil, sediment, sludge, compost, manure, rhizosphere	Bacteria, fungi, algae, nematodes	PCR/qPCR/NGS
Norgen Soil DNA	0.25 g	Bead tube + chemical	Soil with humic acids	Bacteria, fungi, algae	PCR
Automated magnetic bead workflow	0.40 g	Bead-beating + magnetic beads	Agricultural soils with varying textures and organic matter	Bacteria, fungi, archaea	PCR/qPCR/NGS; high-throughput screening

## Data Availability

No new data were created or analyzed in this study.

## Abbreviations

The following abbreviations are used in this manuscript:

16S rRNA	16S ribosomal RNA
2D-GE	two-dimensional gel electrophoresis
ABC	ATP-binding cassette
ACC	1-aminocyclopropane-1-carboxylic acid
ABA	abscisic acid
AMF	arbuscular mycorrhizal fungi
ARGs	antibiotic resistance genes
ASVs	Amplicon Sequence Variants
AutoML	automated machine learning
CI	continuous integration
CCA	canonical correlation analysis
CRISPR	clustered regularly interspaced short palindromic repeats
CRISPR-Cas	clustered regularly interspaced short palindromic repeats–CRISPR-associated systems
Ct	cycle threshold
DADA2	Divisive Amplicon Denoising Algorithm 2
DCL	Dicer-like protein
ddPCR	droplet digital PCR
DIABLO	Data Integration Analysis for Biomarker discovery using Latent cOmponents
dPCR	digital PCR
eggNOG	evolutionary genealogy of genes: Non-supervised Orthologous Groups
ET	ethylene
FAIR	Findable, Accessible, Interoperable and Reusable
GNNs	graph neural networks
GO	Gene Ontology
GRNs	gene regulatory networks
GTDB	Genome Taxonomy Database
HTS	high-throughput sequencing
HSI	hyperspectral imaging
IAA	indole-3-acetic acid
ISA	Investigation/Study/Assay
ISR	induced systemic resistance
ITS	internal transcribed spacer
JA	jasmonic acid
JFA	joint factor analysis
KEGG	Kyoto Encyclopedia of Genes and Genomes
LAI	leaf area index
LFQ	label-free quantification
LUPINE	LongitUdinal modelling with Partial least squares regression for NEtwork inference
MetaCyc	MetaCyc metabolic pathway database
MetaPhlAn	Metagenomic Phylogenetic Analysis
MIAME	Minimum Information About a Microarray Experiment
MIAPE	Minimum Information About a Proteomics Experiment
MIQE	Minimum Information for Publication of Quantitative Real-Time PCR Experiments
MIMARKS	Minimum Information about a Marker Gene Sequence
MIxS	Minimum Information about any (x) Sequence
MOFA	Multi-Omics Factor Analysis
narG	nitrate reductase alpha subunit gene (nitrate reduction)
NCBI	National Center for Biotechnology Information
nifH	nitrogenase iron protein gene (nitrogen fixation)
PCR	polymerase chain reaction
PCA	principal component analysis
PGPMs	plant growth-promoting microbes
PGPR	plant growth-promoting rhizobacteria
PlantTFDB	Plant Transcription Factor Database
PLS	partial least squares
PLS-DA	partial least squares discriminant analysis
POD	peroxidase
PSFs	plant–soil feedbacks
qPCR	quantitative real-time PCR
QIIME 2	Quantitative Insights Into Microbial Ecology 2
QTLs	quantitative trait loci
RDP	Ribosomal Database Project
RefSeq	NCBI Reference Sequence (RefSeq) database
RO-Crate	Research Object Crate (for packaging research data)
RTL	RefSeq Targeted Loci
SA	salicylic acid
SAR	systemic acquired resistance
scRNA-seq	single-cell RNA sequencing
SILVA	SILVA rRNA gene database
siRNAs	small interfering RNAs
SOD	superoxide dismutase
SynComs	synthetic microbial communities
UAV	unmanned aerial vehicle
UNITE	UNITE database for molecular identification of fungi
VOCs	volatile organic compounds
WGCNA	Weighted Gene Co-expression Network Analysis
